# Clinical Profi le and Predictors of Outcomes of Patients with Peripartum Cardiomyopathy: The Philippine Heart Center Experience

**DOI:** 10.7603/s40602-016-0009-0

**Published:** 2016-11-25

**Authors:** Lucky R. Cuenza, Normita Manapat, Jundelle Romulo K. Jalique

**Affiliations:** 1 Clinical Fellow in Cardiology, Philippine Heart Center and National Heart Centre, East Avenue, Quezon City, Philippines 1100; 2 National Heart Centre, Singapore, Singapore; 3 Department of Emergency and Ambulatory Services, Philippine Heart Center, Quezon City, Philippines; 4 Department of Education Training and Research, Philippine Heart Center, Quezon City, Philippines

**Keywords:** peripartum cardiomyopathy, outcomes

## Abstract

**Background:**

Peripartum cardiomyopathy is a rare form of dilated cardiomyopathy characterized by heart failure and left ventricular dysfunction associated with pregnancy. While clinical characteristics of these patients have been previously described in literature, there is limited data regarding the natural history and predictors of outcomes of these patients in Asia, most specifi cally in Filipino patients.

**Methods:**

Clinical and echocardiographic data of 39 patients diagnosed with peripartum cardiomyopathy were analyzed. Patients were followed up for the occurrence of death and major adverse events (MAE) and outcomes were correlated with patient variables.

**Results:**

The mean age of the patients was 28.4 ± 6.9 and the mean ejection fraction (EF) was 27.8 ± 8.4%. Heart failure was the most common symptom (98%) while arrhythmia was the initial presentation in 5 patients (12.8%). 14 patients had recovery of ejection fraction in 6 months (39%) with a mean EF of 55.5 ± 6.3. 16 patients had an initial EF of <25% (41%) and only 2 patients in this subgroup experienced improvement in EF. 29 patients experienced death and/or MAEs (74.4%). Multivariate analysis showed that an EF of <25% (HR 12.0,p=0.019), recovery of LV function (HR 0.23,p=0.05) and improvement of EF in 6 months (HR 0.32,p=0.024) were signifi cant predictors of MAEs. Kaplan Meier curves showed that patients whose ejection fraction was <25% had a 50% incidence of MAEs in 1 year with an increasing trend. Patients whose EF recovered in 6 months experienced a 60% freedom from MAE for almost 6 years. Patients with an EF of <25% had a mortality rate of 50% in two years. Patients with an EF of >25% had a 90% likelihood of survival for 8 years with a higher trend of mortality for patients whose EF did not recover in 6 months.

**Conclusion:**

Peripartum cardiomyopathy is associated with signifi cant morbidity and mortality. The degree of left ventricular dysfunction on presentation as well as improvement of EF within 6 months were predictive for the occurrence of death and major adverse events. This study emphasizes the need for aggressive treatment as well as clinical and echocardiographic follow up early in the course of disease in order to improve outcomes.

## Introduction

Peripartum cardiomyopathy (PPCM) is arbitrarily defi ned as an idiopathic cardiomyopathy presenting with heart failure secondary to left ventricular systolic dysfunction during the duration of pregnancy or in the months following delivery, where no other cause of heart failure is found^1^. The exact cause is unknown but may be related to an interplay of genetic, hormonal, infl ammatory and autoimmune mechanisms^2^. Samonte et al^3^ reported an incidence of 1 in 1270 live births while Lim and colleagues^4^ reported an incidence of 0.89 per 1,000 live births and there is considerable variability across different geographical regions in the world. This condition has been associated with signifi cant morbidity and mortality. While the clinical profi les of these patients have been previously described^3^, there is scarcity of data regarding outcomes of patients in the Asian population, in particular Filipino patients. The objectives of this study were to defi ne the clinical characteristics of these patents in our institution and identify risk factors for the occurrence of major adverse events.

## Methods

This was a retrospective cohort study done at the Philippine Heart Center in patients who were diagnosed with PPCM. The study was conducted after ethical approval was received from the Philippine Heart Center Institutional Ethics Review Board. Relevant information was derived through review of outpatient department records and admissions from 2005 to 2015 along with patient or relative interview and follow up via telephone inquiry. Records of delivery as well as subsequent followup encounters were reviewed for clinical and demographic information. The diagnosis of peripartum cardiomyopathy was defi ned based on the following criteria^5^ 1.Development of congestive heart failure during pregnancy or the fi rst 5 months after delivery; 2.Absence of an identifi able cause for cardiac failure; 3. Absence of recognizable heart disease before pregnancy; and 4. Left ventricular systolic dysfunction with left ventricular ejection fraction (LVEF)<45%. The LVEF during the time of diagnosis was assessed at baseline and on follow up. An EF of >50% was considered to have recovered EF.

### Outcomes

The outcomes were either the occurrence of death and or major adverse events. Major adverse events (MAEs) were defi ned as the occurrence of death and/or complications that were life threatening or resulted in signifi cant morbidity (cardiogenic shock requiring inotropic support, pulmonary edema, arrhythmia that may or may not require either an intracardiac defi brillator or pacemaker and thromboembolism). Mortality data was obtained using the National Statistics Offi ce Index and confi rmed by follow up and chart review, and December 2015 was used as the last follow up date. The time frame of the occurrence of the MAEs was also noted.

### Statistical Analysis

Continuous variables are presented as mean ± standard deviation, and categorical variables as percentages. Kaplan-Meier curves for occurrence of mortality and major adverse events were constructed for the entire cohort. The time-to-event survival models were discrete time models. Similar to the study by Habli et al6, left ventricular ejection fraction was used a predictor of risk with a cutoff of 25% and plotted it with the survival curves along with the recovery of ejection fraction. Variables related to the hazard (risk) of an event were assessed using stepwise multivariable Cox proportional hazards models. All tests were two-sided, and a p value of less than 0.05 was considered statistically signifi cant. All analyses were performed using STATA version 13.0 for Windows(STATA Corp LP, Texas).

## Results

**Table 1:** 

Characteristic (n=39)	Total n(%) or ±SD
Age	28.4 ± 6.9
Parity	1.8 ±1.4
Age >30 years	20 (51.2%)
Hypertension/preeclampsia	13 (33%)
Smoking	2 (5.1%)
Primigravida	23 (58.9%)
Multigravid	16 (41%)
Twin pregnancy	1 (2.5%)
Tocolytic therapy	10 (25.6%)
Symptoms postpartum	37 (94.8%)
Caesarian Section	13 (33.3%)
Normal Delivery	26 (66.7%)
Heart Failure on presentation	37 (98%)
Arrhythmia on presentation	5 (12.8%)
Thromboembolic event on presentation	2 (5.1%)
Ejection Fraction on presentation	27.8 ± 8.4
LVEDD	6.0 ± 1.2
Fractional Shortening	16.8 ± 7.3
LV thrombus	5 (13.2%)
Initial EF <25%,	16 (41%)
Recovery of LV function (>50%)	14 (39%)
Recovery of LVEF in 6 months	13 (36.1%)

LVEDD=Left Ventricular End Diastolic Dimension

EF=Ejection Fraction

LVEF=Left Ventricular Ejection Fraction

**Figure 1:**
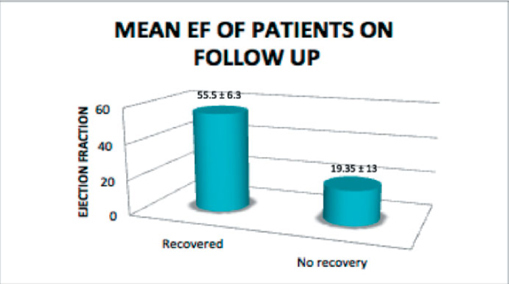

**Figure 2:**
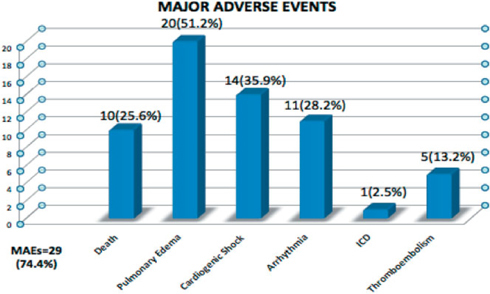

**Table 2:** 

**Univariate Predictors**	**Hazard ratio (95% CI)**	**P value**
Initial EF	0.92 (0.84-0.99)	0.033
Fractional Shortening	0.88(0.79-0.98)	0.024
Arrhythmia on initial presentation	3.38 (1.15-9.93)	0.026
**Multivariate Predictors**	**Hazard ratio (95% CI)**	**P value**
EF <25%	12.0 (1.51-95.5)	0.019
Recovery of LV function	0.23 (0.05-1.01)	0.05
Recovery of LV function in 6 months	0.32 (0.11-0.86)	0.024

**Figure 3:**
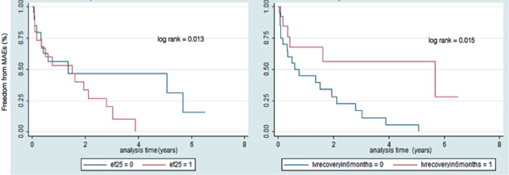

**Figure 4:**
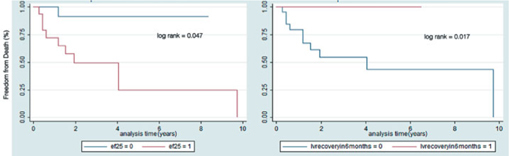

The clinical characteristics of the patients are shown in Table 1. The mean age was 28.4 ± 6.9. The mean parity was 1.8 ±1.4. Symptoms and diagnosis were established postpartum in 37 (94.8%) patients. History of hypertension in pregnancy was noted in 13 (33%) patients. There was only one patient who had a twin pregnancy. 13 (33.3%) underwent caesarian section. Majority of patients presented with heart failure (98%) The mean initial ejection fraction (EF) was 27.8 ± 8.4. The mean left ventricular end diastolic dimension (LVEDD) was 6.0 ± 1.2mm and the mean fractional shortening is 16.8 ± 7.3. There was note of left ventricular thrombus in 5 (13.2%) patients. 16 (41%) had an initial EF of less than 25%, 14 (39%) had recovery of EF and 13 (36.1%) had a demonstrated recovery within 6 months. For patients who recovered the mean EF at recovery was 55.5 ± 6.3. Only 2 patients with an initial EF of <25% exhibited recovery in 6 months (14%). Their mean EF on follow up echocardiogram was 19.35 ± 13 (Figure 1).

There were 29 patients (74.4%) who experienced major adverse events (Figure 2). 10 (25.6%) patients died, 20 (51.2%) had pulmonary edema, 14 (35.9%) had cardiogenic shock, 11 (28.2%) experienced an arrhythmia, and one patient underwent implantation of an intracardiac defi brillator. There were 5 patients who experienced a thromboembolic event (13.2%)

Univariate and multivariate analyses of predictors of major adverse events are listed in Table 2. An Ejection Fraction of <25% (HR 12.0,p=0.019), failure of LV function to recover (HR 0.23,p=0.05) and recovery of LV function within 6 months (HR 0.319,p=0.024) were statistically signifi cant predictors of MAEs on multivariate analysis.

Median follow up was 4.5 years (range 0.15-9.8). The majority occurrence of major adverse events occurs during the fi rst few weeks of diagnosis, and Kaplan Meier curves showed that up to 50% of adverse events occurred within the fi rst year among patients whose EF did not recover. Patients with an ejection fraction of less than 25% who experienced MAEs also had an occurrence of around 50% in 1 year and an increasing incidence of MAEs for 4 years. Patients whose ejection fraction recovered in 6 months experienced a 60% freedom from MAE for almost 6 years while those who did not exhibit recovery were only 25% free from MAEs in the fi rst 2 years (Figure 3). The occurrence of death was noted to be highest in the fi rst two years before reaching an almost 60% free incidence from mortality. Plotting the survival analysis using the level of EF and recovery in 6 months, patients with an ejection fraction of less than 25% had a mortality rate of 50% up to two years. Patients with an ejection fraction of >25% had a good likelihood of survival (90%) continuing for about 8 years. Recovery of ejection fraction also follows a similar trend, with patients whose EF has recovered in 6 months were alive for almost 7 years while patients whose EF did not recover had a signifi cant trend of mortality of around 50% in 4 years (Figure 4).

## Discussion

This study describes the clinical profi le of Filipino patients with peripartum cardiomyopathy and defi nes the predictors of complications. The mean age of patients in our study was 28.4 ± 6.9 which is similar with the study by Elkayam et al, who reported an age range of 28-33 years old with >60% of presenting above 30 years old^7^. In our study the 51.8% presented above the age of 30 years old. And while older age is associated with increased incidence of peripartum cardiomyopathy, the relationship between PPCM and age is not clear. A study by Gentry et al^8^ did not identify maternal age as a risk factor, and the results in our study did not show an association with its occurrence as well as that of adverse events. 13 (33%) patients were hypertensive and 16 (41%) were multigravid. Heart failure is by far the most common presentation, present in 37 of our patients. The other presentations include 5 patients presenting with arrhythmia and 2 patients with a thromboembolic event, 1 patient with stroke and one with peripheral arterial embolism. It should be also noted that 5 patients had left ventricular thrombus by echocardiogram, and these manifestations may refl ect the increased incidence of thromboembolism^9^ as well as the hypercoagulable state associated with pregnancy^10^.

With regard to echocardiographic characteristics, the mean initial EF on presentation was 27.8 ± 8.4 and this was predictive of mortality outcomes. Pooled international data suggested that greater than 50% of patients will demonstrate recovery of LV function^11^. In our study recovery of LV function was present in 39% of subjects with 36% recovery in 6 months. This fi nding is also consistent with literature where the initial degree of myocardial insult is predictive of the recovery of left ventricular function and therefore subsequent events 12.The recovery of ejection fraction as well as recovery within 6 months have been implicated as markers of worse survival and this seems to be consistent in our study^13^ Predictors of LV recovery were not analyzed in our paper, however it should be noted that only 2 patients with an EF <25% exhibited full recovery. In the IPAC study, an LVEF of >30% was predictive of recovery LV function^14^.

The time to event curves gives us an interesting insight with regard to the course of the disease. Most of the adverse events, including death occurred during the acute presentation. 50% of MAEs occurred in the fi rst year and 75% of deaths occurred within the fi rst two years. 29 (74.4%) patients experienced adverse events while 10 (25.6%) died. Similarly, Whitehead et al reported on 17 cases of death due to PPCM between 1991 and 1997 showing an 18% mortality occurring within 1 week and 87% within 6 months of diagnosis^15^. Goland et al reported 182 patients with PPCM. 25% had MAEs with 80% occurring during the fi rst 6 months from diagnosis. He identifi ed EF <25% and failure to recover being associated with these events^16^. In our study there was a decreasing trend of events on follow up as the ejection fraction improved. A low baseline EF may mean a decreased contractile reserve with a decreased likelihood of of recovery^17^ thus predisposing the patient to more adverse events. Indeed it has been shown that severe and persistent LV dysfunction is associated with worse outcomes^18^. The occurrences of death and adverse events in our study and the course of the disease depending on the clinical and echocardiographic parameters on follow up for many years underscore the need for a more aggressive approach in the early management of these patients. Our study showed that patients with an Ejection Fraction of 25%, lack of LVEF recovery and persistent LV dysfunction within 6 months have a higher likelihood of events. Therefore these patients require vigilance of treatment with meticulous clinical and echocardiographic monitoring and follow up^19^. With adequate management there will be responders whose EF will gradually improve^20^, and with it their prognosis and long term outcome can be improved as well.

Our study has certain limitations. First the retrospective nature of our study may affect quality and completeness of data. The single center aspect as well as the rarity of the disease limiting sample size of the study may decrease the power of the statistical test in capturing signifi cant predictors. Also as this is based on a registry, one of the main problems in the diagnosis of peripartum cardiomyopathy is distinguishing PPCM from patients with pre existing dilated cardiomyopathy raising issues of referral bias in the selection of the patient cohort. Ideally the authors would like to suggest a national or even a regional multicenter registry pooling all clinical data in order for us to better elucidate other prognostic variables, which can help in the management of these patients.

Finally, while we can assume that our patients received guideline directed therapy for heart failure, we cannot establish if they were taking optimal doses. Additionally heart transplantation and intracardiac defi brillator devices have been used more frequently in other more developed countries, which may potentially affect outcomes. These two modalities were either not done in our patients or are very infrequently used in our PPCM patients in our country due to socio economic factors and possibly also due to clinicians not being aware of the proper indications of these interventions in patients with PPCM.

To the best of our knowledge this is the fi rst study in our setting, not just in the Philippines but also in Southeast Asia, which not only described the characteristics but also defi ned the clinical course and predictors of outcomes in these patients. The results are consistent and comparable with previous reviews of literature21 where clinical and echocardiographic data can infl uence outcomes. It adds to our knowledge regarding the course of the disease as well as the factors implicated in the development of complications and survival where prompt intervention and surveillance is of paramount importance. The information is useful most especially to guide clinicians in the management and prognosis of these patients.

## Conclusions

Peripartum cardiomyopathy is a rare disease associated with signifi cant morbidity and mortality. The degrees of left ventricular dysfunction on presentation, as well as recovery of EF within 6 months were predictive for the occurrence of major adverse events. This study emphasizes the need for aggressive management and serial clinical and echocardiographic monitoring in the course of the disease in order to improve outcomes.
